# Modified Pulsatilla decoction ameliorates ulcerative colitis by affecting gut microbiota and metabolite profiles

**DOI:** 10.3389/fmicb.2025.1582559

**Published:** 2025-05-12

**Authors:** Zhixin Fu, Xiangyue Xie, Yulei Wang, Zhen Wang, Anran Wu, Shaotang Ye, Yongbo Liu

**Affiliations:** ^1^College of Animal Science and Technology, Hebei Normal University of Science & Technology, Qinhuangdao, China; ^2^Hebei Key Laboratory of Veterinary Preventive Medicine, College of Animal Science and Technology, Hebei Normal University of Science & Technology, Qinhuangdao, China; ^3^Faculty of Veterinary Medicine, Chiangmai University, Chiangmai, Thailand; ^4^College of Veterinary Medicine, South China Agricultural University, Guangzhou, China; ^5^Henry Fok School of Biology and Agriculture, Shaoguan University, Shaoguan, China

**Keywords:** modified Pulsatilla decoction, ulcerative colitis, 16S rRNA sequencing, gut microbiota, metabolomics

## Abstract

To investigate the therapeutic effects of a specific modified Pulsatilla decoction (MPD) on ulcerative colitis (UC) in mice, 32 male Balb/c mice were randomly assigned to four groups: Control, Model, High-dose (H-dose), and Low-dose (L-dose), with eight mice per group. All groups except the Control group were administered 3% dextran sulfate sodium (DSS) in their drinking water for 7 days to induce acute UC. The H-dose group and L-dose group mice were gavaged, respectively, with different concentrations of MPD, while the Control group and Model group received the same amount of steriled water by gavage. Clinical symptoms of the mice were observed and recorded throughout the study. Subsequently, pathological sections of the colon tissues were prepared, and 16S rRNA high-throughput sequencing and metabolomics analysis were conducted on the intestinal contents. The results indicated that MPD improved the structure and morphology of colon tissue, significantly reducing inflammatory damage in DSS-treated mice. Furthermore, MPD alleviated DSS-induced intestinal injury by enhancing the abundance of beneficial intestinal probiotics, such as *Actinobacteriota* and *Oscillospirates*. Metabolomic analysis revealed significant changes in the MPD group compared to the Model group, with 53 metabolites upregulated and 22 downregulated. Key upregulated metabolites included Esculetin, Glutarate semialdehyde, and Licoricone, while downregulated metabolites included Ectoine and Trans-Piceid. KEGG enrichment analysis indicated that MPD primarily targets pathways such as linoleic acid metabolism, VEGF signaling, and glutamatergic synapse, highlighting its potential regulatory effects. In conclusion, we revealed that this MPD has the potential to alleviate DSS-induced colitis by reducing inflammation, regulating intestinal microbiota and intestinal metabolism.

## 1 Introduction

Epidemiological studies indicate a rising global incidence of ulcerative colitis (UC) each year (Vadstrup et al., 2020). UC is a chronic, non-specific inflammatory bowel disease with an unclear pathogenesis (Mehrmal et al., 2021). Contributing factors to UC include environmental influences, genetic predisposition, alterations in intestinal microbiota, and immune responses (Annese, 2020). The disease is characterized by recurrent episodes and difficulties in achieving healing (Borren et al., 2021). Common clinical symptoms include diarrhea, fever, abdominal pain, bloody stools, loss of appetite, and weight loss. Furthermore, persistent inflammation of the rectal and colonic mucosa can elevate the risk of developing colon cancer (Feuerstein et al., 2019).

Current treatment options of Western Medicine primarily consist of aminosalicylic acids, corticosteroids, and immunomodulatory drugs. However, these therapies often exhibit limited efficacy, significant side effects, and poor tolerance (Wu et al., 2020). In contrast, traditional Chinese medicine (TCM) represents a treasure trove of human medical heritage. TCM is distinguished by its ‌multi-target effects‌, ‌synergistic interactions of multiple bioactive components‌ and low side effects, thereby offering unique advantages in managing UC.

Recent updates in evidence-based medicine indicate that TCM may be effective in the treatment of UC, providing advantages such as reduced toxicity and fewer side effects, as well as a decreased likelihood of symptom recurrence (Yang et al., 2020; Zhang et al., 2024). In clinical formulations of TCM, *Magnolia officinalis* is utilized for the treatment of respiratory and digestive diseases. *Magnolol*, a principal component of this botanical drug, exhibits anti-inflammatory, antioxidant, anti-tumor, and cell-protective effects (Chen et al., 2019). Research has demonstrated that *magnolol* can alleviate colonic lesions, protect the intestinal mucosa, and reduce diarrhea in mouse models of UC (Chen et al., 2021). *Lonicera hypoglauca* has shown anti-tumor activity against human lung cancer cells and possesses anti-allergic properties (Chan et al., 2008; Leung et al., 2008). Modern studies suggest that *Terminalia chebula* may benefit UC treatment by down-regulating pro-inflammatory factors in the colonic mucosa (Dong and Xue, 2014; Wen et al., 2019). *Scutellaria baicalensis* is widely employed in the treatment of diarrhea, hypertension, dysentery, and hemorrhaging (Liao et al., 2021). Additionally, a recent study found that *Zingiberis rhizoma* decoction can reduce serum inflammatory factors in UC mice and protect their intestinal structure (Chen et al., 2023).

Baitouweng decoction, originating from the *Shang Han Lun*, consists of Pulsatilla root (*Baitouweng*), Coptis root (*Huanglian*), Phellodendron bark (*Huangbai*), and Fraxinus bark (*Qinpi*). It is traditionally used to clear heat, detoxify, cool the blood, and alleviate dysentery caused by damp-heat, with clinical manifestations such as abdominal pain, bloody diarrhea, and tenesmus. However, due to the cold nature of its herbs, improper use may deplete healthy qi (vital energy). Building on this foundation and incorporating clinical experience, we developed the ‌modified Pulsatilla decoction, which effectively arrests diarrhea while preserving the body’s vital energy. Based on Baitouweng decoction, additional botanical drugs were incorporated, including *Rhubarb*, *Curcuma*, *Gardenia*, *Scutellaria*, *Terminalia chebula Retz*, *White Peony Root*, and *Ginger Powder* (Zhang et al., 2023). These additions enhance the formula’s effects by strengthening heat-clearing, dampness-drying, and blood-cooling properties, while also providing liver-soothing, pain-relieving, and intestinal-regulating functions. This modified formula is designed to address damp-heat dysentery and inflammatory conditions more comprehensively (Li et al., 2014; Song et al., 2021; Xu et al., 2012).

This study examines a specific ‌modified Pulsatilla decoction (MPD) composed of Pulsatilla Chinensis (Bunge) Regel, *Magnolia officinalis*, *Honeysuckle*, *Terminalia chebula Retz*, *Scutellaria baicalensis*, *Ginger Powder*, *Phellodendron chinense*, *Coptis chinensis*, *Cortex Qinpi*, *Rhubarb*, *Radix Curcumae*, and *Gardenia jasminoides*, as well as *White Peony Root*, etc. TCM compounds, which comprise multiple ingredients, provide a multi-targeted therapeutic approach and several advantages (Mai et al., 2019). This study aims to evaluate the efficacy of the MPD in alleviating symptoms of UC in mice, specifically by protecting intestinal tissue and modulating intestinal flora. We believe that this study will provide a data foundation for the development of traditional Chinese medicine formulations for the treatment of UC, and has research value in traditional Chinese medicine.

## 2 Materials and methods

### 2.1 Composition and preparation of MPD

MPD is a Chinese herbal mixture composed of *Pulsatilla Chinensis (Bunge) Regel* [Ranunculaceae; *Pulsatillae radix*] (15 g), *Magnolia officinalis* [Magnoliaceae; *Magnoliae Officinalis Cortex*] (15 g), *Honeysuckle* [Caprifoliaceae; *Lonicerae Flos*] (5 g), *Terminalia chebula Retz* [Combretaceae; *Terminaliae Fructus*] (5 g), *Scutellaria baicalensis* [Lamiaceae; *Scutellariae radix*] (10 g), *Ginger Powder* [Zingiberaceae; *Zingiberis Rhizoma Recens*] (5 g), *Phellodendron chinense* [Rutaceae; *Phellodendri Chinensis Cortex*] (15 g), *Coptis chinensis* [Ranunculaceae; *Coptidis Rhizoma*] (10 g), *Cortex Qinpi* [Oleaceae; *Fraxini Cortex*] (20 g), *Rhubarb* [Polygonaceae; *Rhei Radix et Rhizoma*] (20 g), *Radix Curcumae* [Zingiberaceae; *Curcumae Radix*] (10 g), *Gardenia jasminoides* [Rubiaceae; *Gardeniae Fructus*] (10 g), and *White Peony Root* [Ranunculaceae; *Paeoniae Alba Radix*] (5 g). These Chinese herbals were sourced from the Anguo Traditional Chinese Botanical Drugs Market in Hebei Province, China. The medicinal materials used in this study have been identified by Traditional Chinese Veterinary Medicine research Laboratory of Hebei Normal University of Science & Technology. The botanical drugs were dried, crushed, and screened through an 80-mesh sieve before being mixed in specific proportions. Based on the aforementioned ratio, 145 g mixture of the crude herbs was soaked in 1,450 mL of water overnight (approximately 10 h), boiled for 2 h, and then filtered. The same procedure was repeated with 750 mL of water. The combined filtrates were concentrated to a crude drug concentration of 1 g crude herbs/mL (low-dose group) and stored at 4°C. A high-dose decoction with a concentration of 2 g crude herbs/mL was prepared following the same method.

### 2.2 Component analysis of MPD with UHPLC-MS/MS

The chemical composition of MPD was analyzed using a Thermo QE Plus liquid chromatography-tandem high-resolution mass spectrometry system, coupled with Compound Discoverer data processing software. The UHPLC-MS/MS analysis was performed on a Q Exactive Plus Orbitrap system (Thermo Fisher Scientific, Bremen, Germany) integrated with an Ultra-High Performance Liquid Chromatography unit (U3000, Thermo Fisher Scientific, Bremen, Germany). Mass spectrometry data were acquired in Full MS-ddMS2 mode using positive and negative ion scans, with a mass-to-charge (*m*/*z*) range of 100–1,200.

The MS1 resolution was configured to 70,000, and MS2 resolution was set at 17,500. Operational parameters included a 3.2 kV ion source voltage, a capillary transfer tube temperature of 320°C, and an auxiliary gas heater temperature of 350°C. Sheath gas flow was maintained at 40 L/min, with auxiliary gas flow at 15 L/min. The automatic gain control (AGC) target was set to 1 × 10^6^, and data-dependent MS2 scans (TopN = 5) were triggered using stepped normalized collision energies (NCE) of 30, 40, and 50. These parameters ensured robust identification of MPD’s chemical constituents.

### 2.3 Animals experimental design and diets

**Figure 1:**
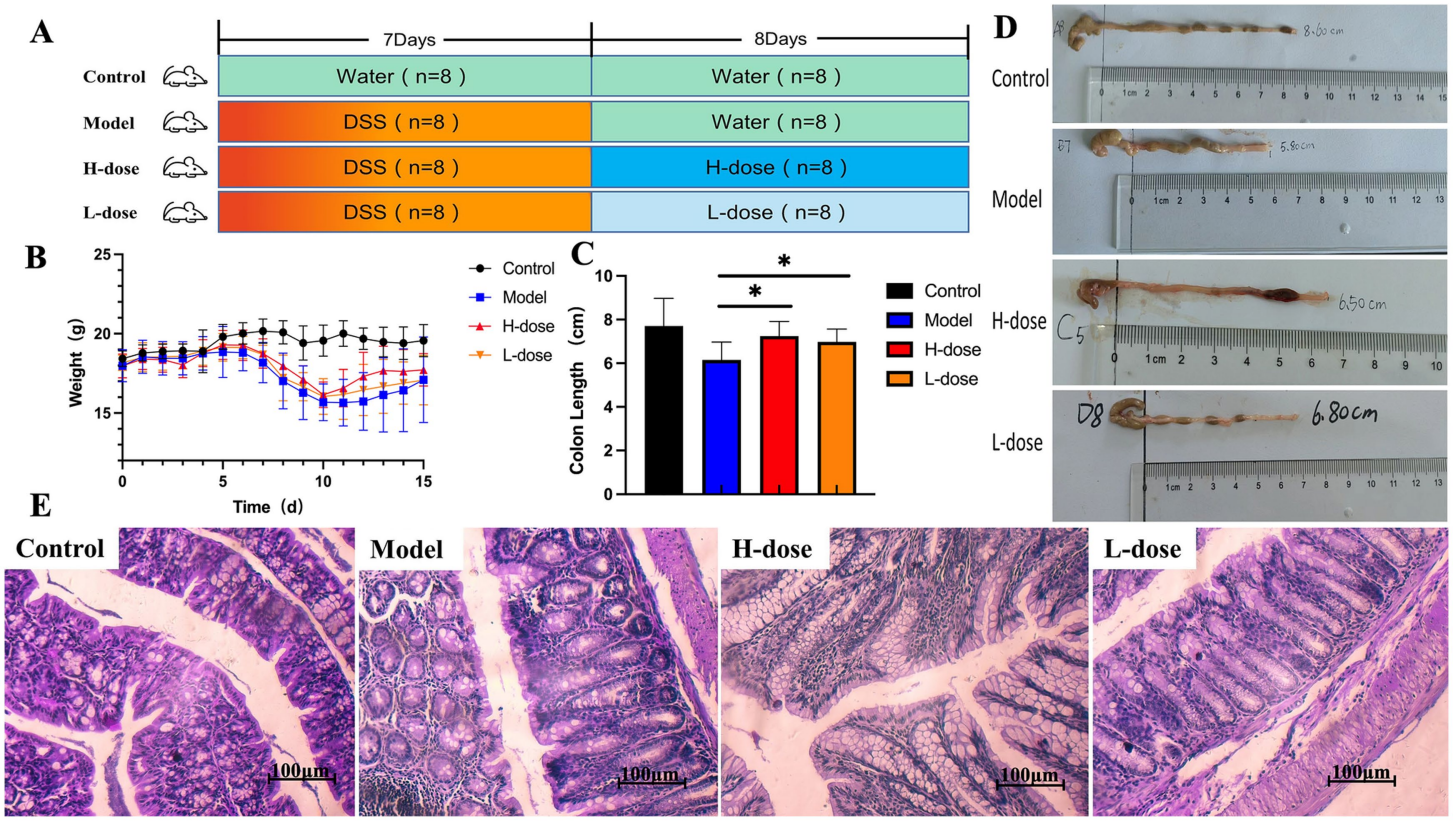
MPD significantly improves colitis symptoms in mice. **(A)** The experimental scheme of MPD treatment in DSS-induced colitis in mice. **(B)** Body weight (*n* = 8). **(C)** Colon length (*n* = 8). **(D)** Representative picture of the colon. **(E)** Representative image of mouse colon stained with H&E (×400). All data are presented as mean ± standard deviation (SD). ^*^p < 0.05, significantly different compared to Model group.

Thirty-two male Balb/c mice (6–7 weeks old, 20 ± 2 g) were purchased from Sibeifu Biotechnology Co., Ltd. (Beijing, China). The animals were housed in a specific pathogen-free facility under controlled conditions: temperature maintained at 23–25°C, relative humidity of 40–60%, and a 12-h light-dark cycle. They had unrestricted access to food and water. Following a one-week acclimation period, the mice were randomly assigned to four groups: the Control group, the Model group, the H-dose group, and the L-dose group. As illustrated in Figure 1A, the Model, H-dose, and L-dose groups were administered a 3% (w/v) DSS solution for 7 days (Chen et al., 2020). Over the following 8 days, each group of mice was normally provided with sterile drinking water. Regarding the dosage of MPD administered, the daily dosage for adults is 145 g of crude herbs, to be consumed after decoction. The equivalent dose of 60 kg for mice and adults was calculated according to the conversion coefficient of body surface area (Wang et al., 2021). Accordingly, mice of L-dose group were administered crude herbs at doses of 10 g/kg (approximately corresponding to half the commonly used MPD dose for clinical patients), and the H-dose group were 20 g/kg (approximately equivalent to the standard MPD dose for clinical patients). According to the gavage protocol of 0.1 mL per 10 g body weight for mice, 0.2 mL of corresponding decoction was administered to both the H-dose and L-dose groups. Meanwhile, 0.2 mL of sterile water was given to the Control and Model groups under the same conditions. The Control group was provided sterile water throughout the experiment. At the conclusion of the treatment period, all mice were fasted for 24 h before being euthanized by cervical dislocation under professional guidance. Efforts were made to ensure the well-being of the mice, minimize suffering, and reduce the number of animals used.

### 2.4 Sample collection

To assess the severity of colitis, we recorded daily body weight measurements of all mice. The colon was isolated and measured for length, and colonic tissue was fixed in 10% (w/v) formalin for subsequent pathological analysis. Fresh feces were collected and stored at −80°C for further analysis.

### 2.5 Histological procedures

Colon tissues were retrieved from formalin, processed for paraffin embedding, sectioned using a microtome, and stained with hematoxylin and eosin (H&E) for histopathological examination.

### 2.6 16S rRNA sequencing and gut microbiota analysis

Microbial DNA was extracted from stool samples using the QIAamp DNA Stool Kit (Qiagen, Valencia, United States). Purification of PCR products was performed with Vazyme VAHTS^™^ DNA Clean Beads (Vazyme, Shanghai, China), and quantification was carried out using the PicoGreen dsDNA Assay Kit (Invitrogen, United States). The V3–V4 region of the 16S rRNA gene was amplified and sequenced using the Illumina MiSeq PE300 platform. Data analysis was conducted via the Majorbio Cloud Platform.^1^

### 2.7 Metabolomics analysis

#### 2.7.1 Chromatographic conditions

A 2 μL sample was separated on an HSS T3 column (100 mm × 2.1 mm i.d., 1.8 μm) before mass spectrometry analysis. The mobile phase consisted of solvent A (0.1% formic acid in water: acetonitrile, 95:5, v/v) and solvent B (0.1% formic acid in acetonitrile: isopropanol: water, 47.5:47.5:5, v/v). The gradient elution program was as follows: 0–0.1 min, 0–5% B; 0.1–2 min, 5–25% B; 2–9 min, 25–100% B; 9–13 min, 100% B; 13–13.1 min, 100–0% B; and 13.1–16 min, 0% B for system equilibration. The injection volume was 2 μL, with a flow rate of 0.4 mL/min, while the column temperature was maintained at 40°C. Samples were stored at 4°C during the analysis.

#### 2.7.2 Mass spectrometry conditions

UHPLC-MS/MS (Ultra-high performance liquid chromatography tandem mass spectrometry) analysis was performed using a Thermo UHPLC-Q Exactive Mass Spectrometer equipped with an electrospray ionization (ESI) source, operating in both positive and negative ion modes. Key parameters included a heater temperature of 400°C, capillary temperature of 320°C, sheath gas flow rate of 40 arb, and auxiliary gas flow rate of 10 arb. The ion-spray voltage was set to −2800 V in negative mode and 3,500 V in positive mode, with normalized collision energy rolling between 20–40–60 V for MS/MS. Full MS resolution was configured at 70,000, and MS/MS resolution at 17,500. Data were acquired in Data Dependent Acquisition (DDA) mode across a mass range of 70–1,050 *m*/*z*.

#### 2.7.3 Identification of metabolites

Accurately weigh 50 ± 5 mg of the sample into a 2 mL centrifuge tube. Add a 6 mm grinding bead and 400 μL of extraction solution (methanol:water = 4:1, v/v) containing four internal standards, including L-2-chlorophenylalanine (0.02 mg/mL). Freeze and grind the sample at −10°C with a frequency of 50 Hz, followed by ultrasonic extraction at 5°C for 30 min. Allow the sample to stand at −20°C for 30 min, then centrifuge it at 13,000 g and 4°C for 15 min. Collect the supernatant for LC-MS analysis under the specified conditions. The raw data was imported into metabolomics processing software, Progenesis QI v3.0 (Waters Corporation, Milford, United States), for baseline correction, peak identification, integration, retention time alignment, and other processes. This produced a data matrix containing retention times, mass-to-charge ratios, and peak intensities. Characteristic peaks were identified using the software, and MS and MS/MS spectrometry data were matched with metabolic databases. A mass error threshold of less than 10 ppm was applied, and secondary mass spectrometry scores were used for metabolite identification. The primary databases referenced included public repositories such as the Human Metabolome Database^2^ and METLIN.^3^

### 2.8 Statistical analysis

Data were organized using Excel and analyzed with SPSS 20 and GraphPad Prism 7.0 software. Statistical differences were assessed using one-way ANOVA followed by Tukey’s multiple range tests, while the Kruskal–Wallis rank sum test was employed for analyzing the relative abundance of gut microbiota. Results are presented as mean ± standard deviation (SD) (*n* = 8). ^*^p < 0.05, significantly different compared to Model group.

## 3 Results

### 3.1 The chemical components of MPD

**Figure 2:**
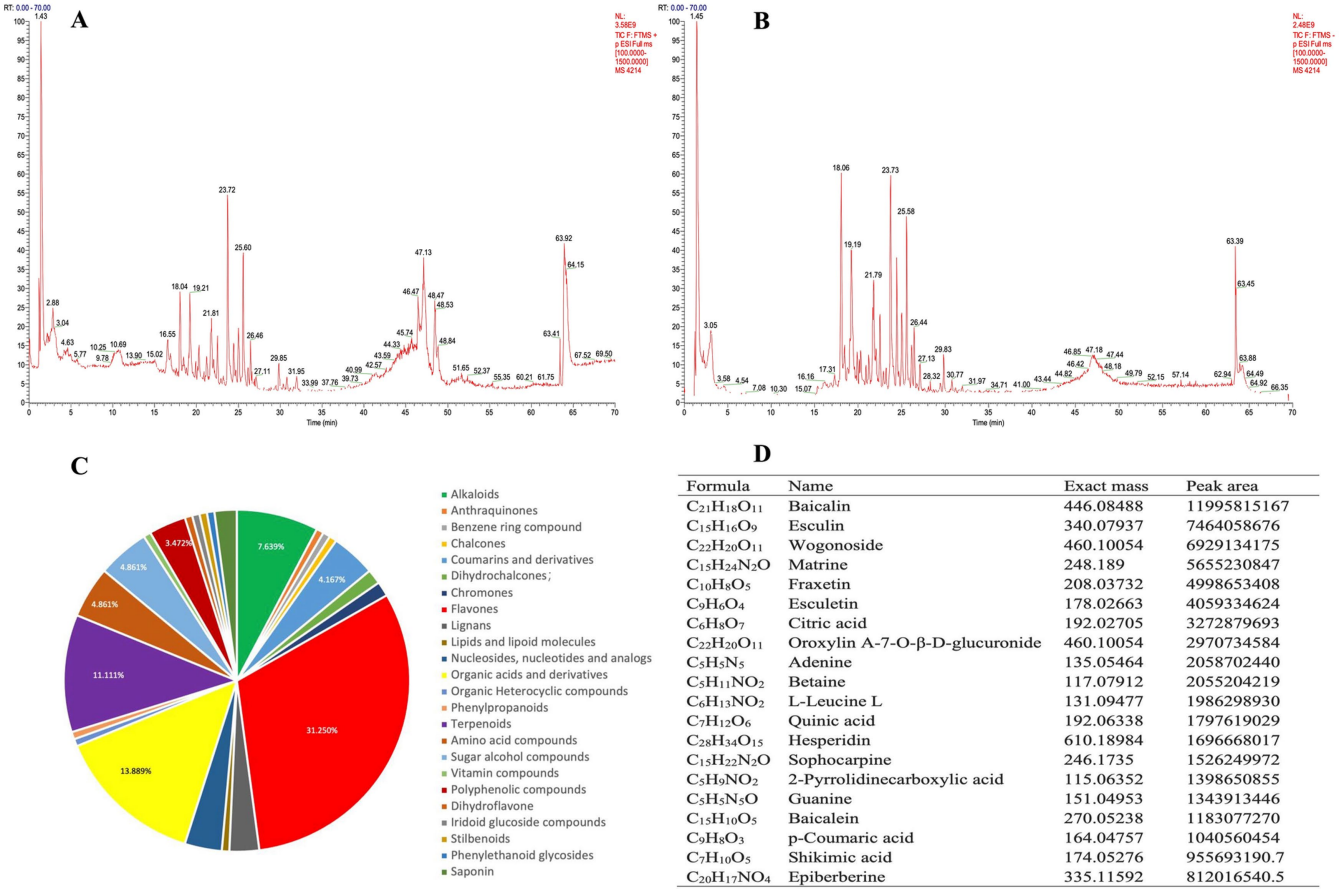
Chemical identification of MPD using UHPLC-MS/MS. **(A)** The chemical total ion chromatogram (TIC) chromatogram of MPD in the positive ion mode. **(B)** The TIC chromatogram of MPD in the negative ion mode. **(C)** Structural classification of compounds contained in MPD. **(D)** Representative chemical components of MPD identified by UHPLC-MS/MS.

The chromatogram of MPD’s total ion chromatogram (TIC) was divided into two groups based on UHPLC-MS/MS’s positive and negative ion modes (Figures 2A,B). Compound discover 3.2 software was used to extract characteristic peaks from the original Raw mass spectrometry data, and the mass deviations of characteristic peak element matching, molecular formula prediction, and isotope distribution matching were all set to within 5 ppm. The mzCloud online database and the locally built mzVault database of natural products of traditional Chinese medicine were used to identify characteristic peaks, and the screening criteria for positive results were mass deviation <5 ppm, consistent with isotope distribution, and mzVault best match database matching score >70 points. A total of 144 compounds were identified, including 45 flavones, 20 organic acids, 16 terpenoids, 11 alkaloids, 7 amino acids, 7 sugar alcohols, 6 coumarins and derivatives, 5 nucleotides and analogs, 5 polyphenolics, 4 lignans, 3 lignans, 2 dihydrochalcones, 2 dihydrochalcones, 1 derivatives, 1 benzene ring, 1 lipoid molecule, 1 organic heterocyclic, 1 phenylpropanoid, 1 vitamin, 1 dihydroflavone, 1 iridoid glucoside, 1 stilbenoid, and 1 phenylethanoid glycoside etc. in MPD (Figure 2C). The top compounds with the largest peak area included Baicalin, Esculin, Wogonoside, Matrine, Fraxetin, Esculetin, Citric acid, Oroxylin A-7-O-β-D-glucuronide, Adenine, Betaine, L-Leucine, Quinic acid, Hesperidin, Sophocarpine, and others (Figure 2D).

### 3.2 Effect of MPD on body weight and colon in mice

Compared to the control group, all DSS-treated mice experienced body weight loss. Notably, the body weight of the H-dose group tended to increase during high-dose MPD administration compared to the Model group (Figure 1B). DSS-treated mice also exhibited shorter colon lengths, but MPD treatment significantly reversed these changes, particularly at higher doses (Figures 1C,D, *p* < 0.05). The crypt structure and morphology of colon tissue were significantly improved in both the H-dose and L-dose groups, with a substantial number of goblet cells observed and reduced inflammatory cell infiltration compared to the DSS group. No obvious abnormalities were found in the intestinal sections of the control group, which displayed an intact epithelial cell layer structure and no inflammatory cell infiltration. In contrast, the model group exhibited extensive inflammatory cell infiltration. The epithelial cell layer in the H-dose and L-dose groups was relatively intact, with fewer inflammatory cells present. There was no significant difference between the H-dose and L-dose groups (Figure 1E).

### 3.3 Effect of MPD on microbiota diversity and composition of stool samples

**Figure 3:**
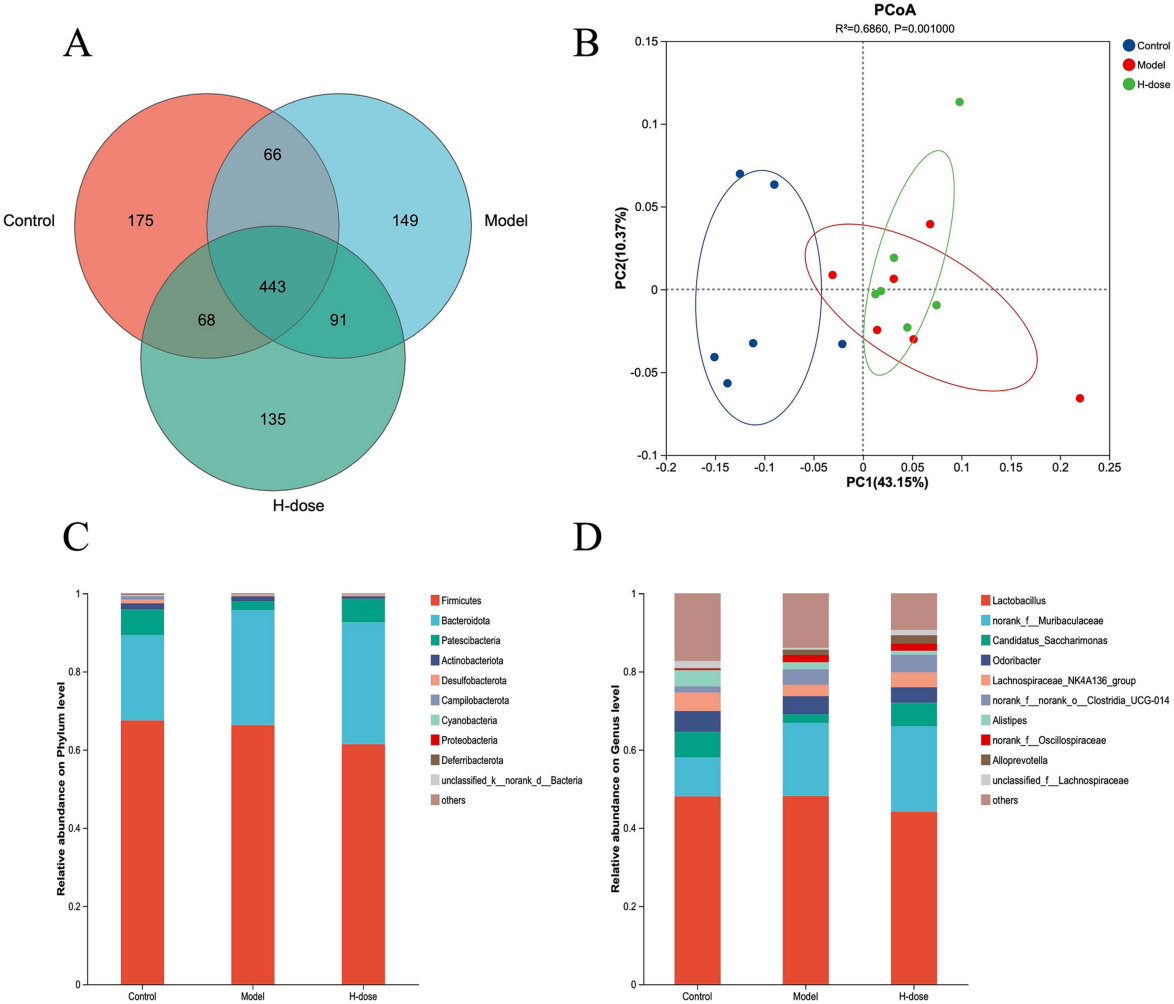
Venn diagram **(A)** of the gut microbiota of mice in different groups. **(B)** PCoA plots derived from the Bray–Curtis dissimilarities of bacterial community composition for each group. The relative abundance of the phylum **(C)** and genus **(D)** taxa of bacteria in each group.

A Venn diagram illustrated the presence of 443 OTUs among the three groups, with 175, 149, and 135 specific OTUs unique to the Control, Model, and H-dose groups, respectively (Figure 3A). Principal coordinate analysis (PCoA) revealed distinct clusters of microbial composition between the Control group and the DSS-treated groups (Figure 3B). These results indicate that DSS induces significant changes in mouse fecal microbiota. We utilized Chao 1, Pielou, ACE, Sobs, Shannon, and Simpson indices to assess gut microbiota alpha diversity (Table 1). The Model and H-dose groups exhibited a significant decrease in Chao 1 and Sobs indices compared to the Control group (*p* < 0.05), while the H-dose group also demonstrated a significantly lower ACE index (*p* < 0.05).

**Table 1:** Alpha diversity analysis of microbiota of mice in different group.

Items	Control	Model	H-dose	*p*-value
Sobs	397.17 ± 30.12^a^	345.17 ± 22.36^b^	336.33 ± 50.25^b^	0.022
Shannon	3.21 ± 0.28	3 ± 0.46	3.1 ± 0.27	0.609
Simpson	0.12 ± 0.05	0.14 ± 0.05	0.12 ± 0.04	0.999
Ace	454.27 ± 31.91^a^	402.28 ± 19.21^ab^	390.3 ± 68.61^b^	0.027
Chao	450.32 ± 27.88^a^	395.07 ± 24.28^b^	382.88 ± 68.06^b^	0.044
Pielou_e	0.50 ± 0.09	0.51 ± 0.08	0.53 ± 0.04	0.739

The lowercase letter “a” represents that there is no significant difference between this group and other groups marked with “a.” The lowercase letter “ab” represents that there is no significant difference between this group and other groups labeled “a,” but there may be significant differences between this group and other groups labeled “b.” The lowercase letter “b” indicates a significant difference between this group and other groups marked with “a.”

**Figure 4:**
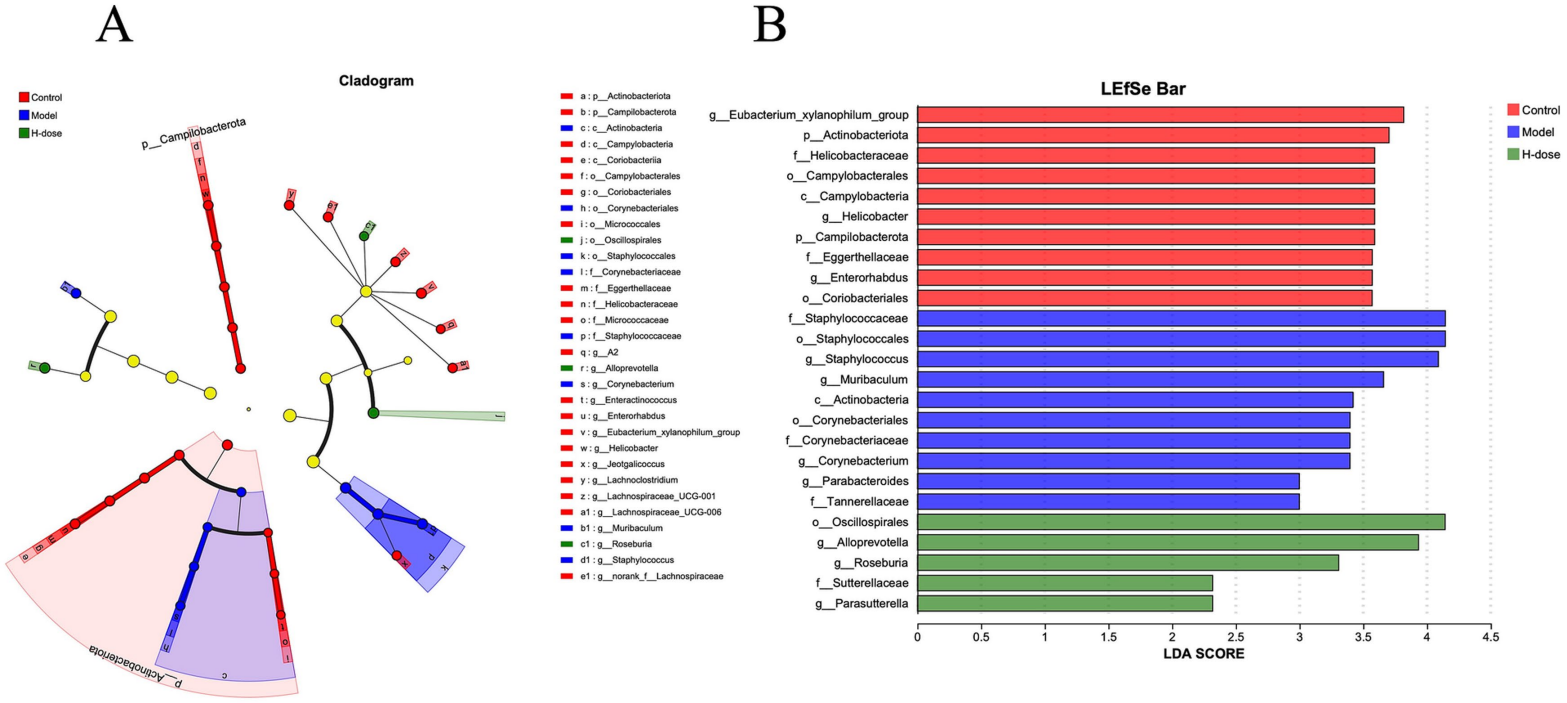
**(A)** Cladograms generated by LEfSe depicting taxonomic associations among the three groups of mice microbial communities. **(B)** An LDA score based on the LEfSe method is shown and highlights the taxonomic groups in the Control, Model, and H-dose group and LDA score >4.

We further analyzed the most dominant microbiota at the phylum level to assess overall shifts in gut microbiota composition among the groups (Figure 3C and Table 2). The Model group exhibited a lower relative abundance of *Actinobacteriota* compared to the Control and H-dose groups (*p* < 0.05). Additionally, both the Model and H-dose groups showed a reduced relative abundance of *Campilobacterota* (*p* < 0.05). Analysis of the seven predominant genera in each group revealed specific changes in microbial taxa (Figure 3D and Table 3). LEfSe analysis identified 21 biomarkers in the Control group, 8 in the Model group, and 4 in the H-dose group, indicating significant reductions in certain species due to DSS, which led to lower relative abundance in the cecal microbiota of the DSS groups (Figure 4A). *Staphylococcus* was predominant in the Model group at the phylum, genus, and class levels (LDA >4), while *Oscillospirates* was more abundant in the H-dose group (LDA >4) (Figure 4B).

**Table 2:** Relative abundance of the seven dominant phyla in each group.

Name of species	Control	Model	H-dose	*p*-value
*Firmicutes*	67.49 ± 12.55	61.41 ± 16.38	66.3 ± 12.42	0.910
*Bacteroidetes*	21.82 ± 9.9	31.19 ± 15.17	29.4 ± 13.68	0.420
*Patescibacteriota*	6.53 ± 5.27	6.03 ± 6.25	2.27 ± 1.81	0.202
*Actinobacteriaota*	1.62 ± 0.37^a^	0.61 ± 0.18^b^	1.26 ± 0.62^a^	0.010
*Desulfobacterota*	0.91 ± 1.21	0.38 ± 0.25	0.35 ± 0.3	0.982
*Campilobacterota*	0.91 ± 0.8^a^	0.05 ± 0.02^b^	0.11 ± 0.08^a^	0.009
*Cyanobacteria*	0.39 ± 0.3	0.17 ± 0.16	0.15 ± 0.07	0.196

The lowercase letter “a” represents that there is no significant difference between this group and other groups marked with “a.” The lowercase letter “b” indicates a significant difference between this group and other groups marked with “a.”

**Table 3:** Relative abundance of the seven dominant genera in each group.

Name of species	Control	Model	H-dose	*p*-value
*Lactobacillus*	48.06 ± 17.63	44.2 ± 12.15	48.17 ± 15.23	0.932
*Norank_f__Muribaculaceae*	9.98 ± 5.4	21.79 ± 11.12	18.65 ± 8.07	0.052
*Candidatus_Saccharimonas*	6.53 ± 5.27	6.03 ± 6.25	2.27 ± 1.81	0.203
*Odoribacter*	5.34 ± 5.31	3.96 ± 2.93	4.62 ± 5.1	0.983
si*Lachnospiraceae_NK4A136_group*	4.72 ± 4.61	3.82 ± 4.32	2.9 ± 3.05	0.641
*Norank_f__norank_o__Clostridia_UCG-014*	1.57 ± 1.11	4.52 ± 2.51	3.98 ± 2.94	0.109
*Alistipes*	4.15 ± 3.19	0.99 ± 0.44	1.77 ± 2.09	0.182

### 3.4 Effect of MPD on intestinal metabolism

**Figure 5:**
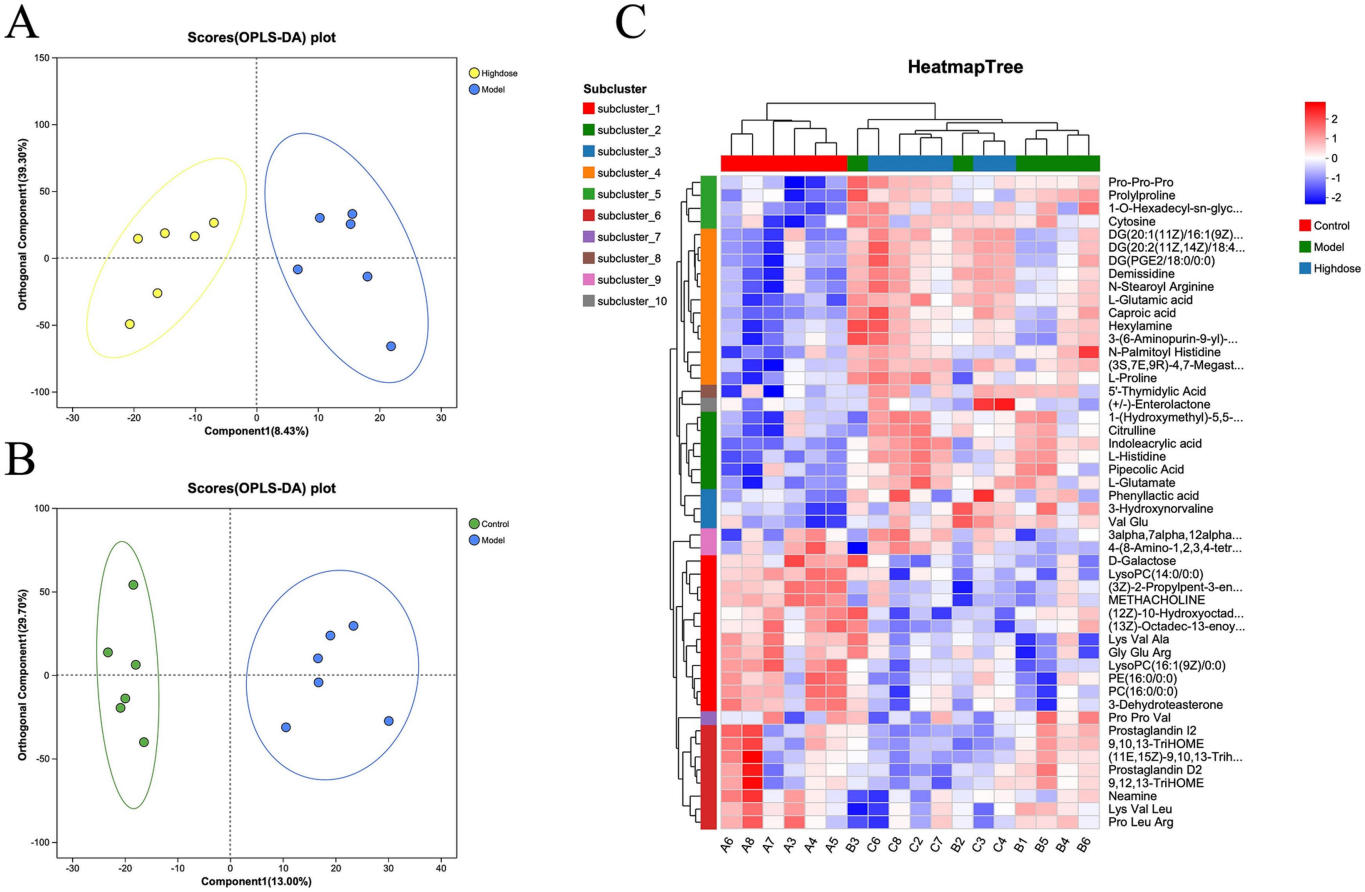
OPLS-DA score plots between Model group and Control group individuals for tissue **(A)** (*R*^2^*Y* = 0.9; *Q*^2^ = 0.78) and H-dose group. **(B)** (*R*^2^*Y* = 0.947 and *Q*^2^ = 0.58). **(C)** Heatmap of metabolite cluster analysis in each group.

Orthogonal partial least squares discriminant analysis (OPLS-DA) demonstrated a clear separation between the Control and Model group samples (Figure 5A), indicating the successful establishment of a DSS-induced ulcerative colitis mouse model. MPD treatment resulted in a distinct separation between the MPD and Model group samples (Figure 5B). Clustering heatmap analysis of differential metabolites revealed that the metabolite profile of the MPD group was similar to that of the Control group (Figure 5C). These results suggest that MPD effectively ameliorates DSS-induced intestinal metabolite disorders in mice.

**Figure 6:**
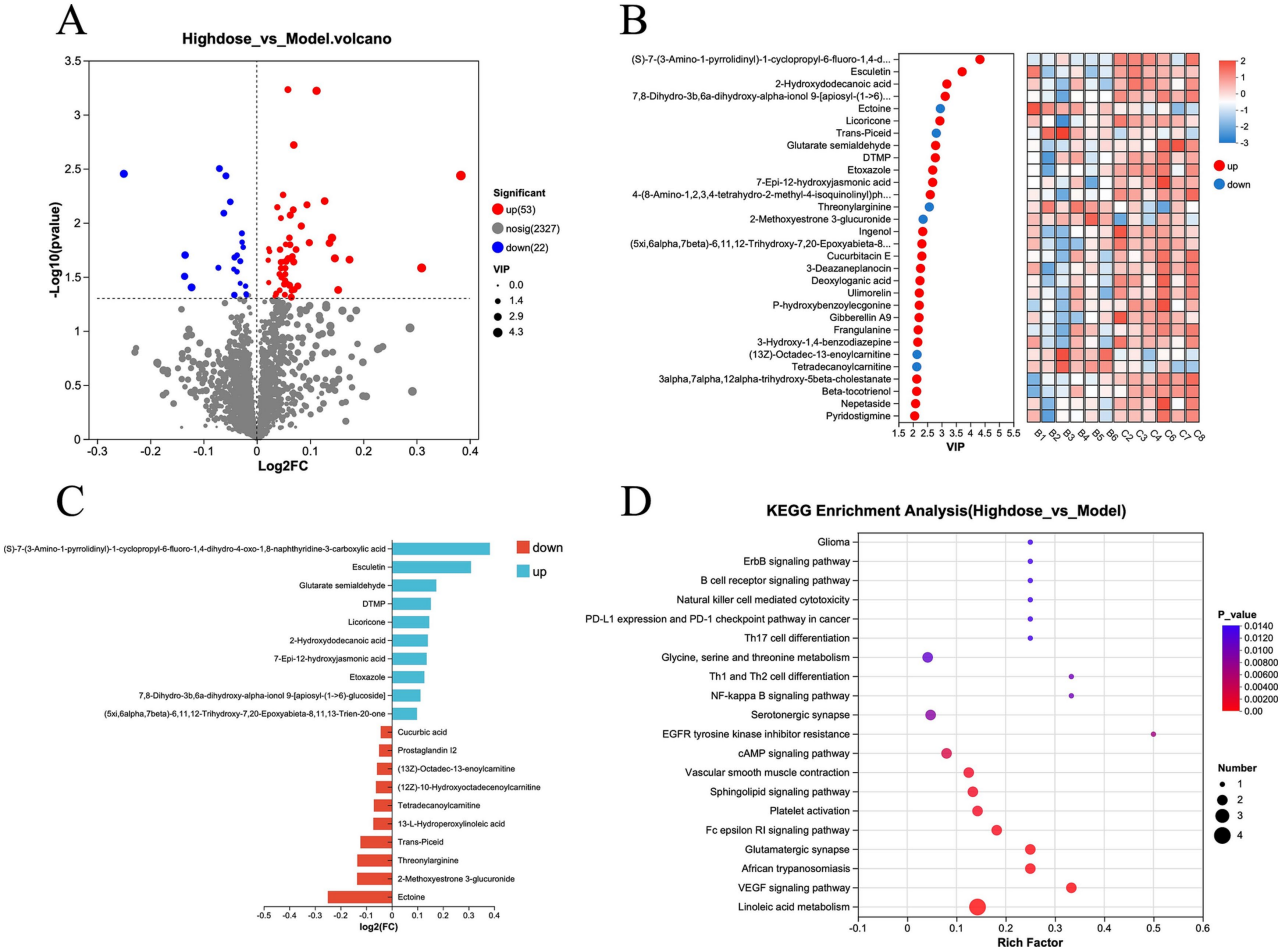
**(A)** Volcano plot of differential metabolites, H-dose group vs. model group. **(B)** Heatmap analysis of variable importance in projection (VIP) values for differential metabolites, H-dose group vs. model group. **(C)** Bar chart of fold change in metabolite differences, H-dose group vs. model group. **(D)** KEGG enrichment analysis of differential metabolites, H-dose group vs. model group.

Further analysis of the metabolite data from the Model and MPD groups revealed that 53 metabolites were upregulated and 22 were downregulated in the MPD group compared to the Model group (Figure 6A). For a more precise analysis, differential metabolites were presented using variable importance in projection (VIP) (Figure 6B). The top five upregulated metabolites with the highest fold changes were (S)-7-(3-Amino-1-pyrrolidinyl)-1-cyclopropyl-6-fluoro-1,4-dihydro-4-oxo-1,8-naphthyridine-3-carboxylic acid, Esculetin, Glutarate semialdehyde, DTMP, and Licoricone. The top five downregulated metabolites included Ectoine, 2-Methoxyestrone 3-glucuronide, Threonylarginine, Trans-Piceid, and 13-L-Hydroperoxylinoleic acid (Figure 6C). KEGG enrichment analysis of these differential metabolites indicated that MPD primarily affects pathways such as Linoleic acid metabolism, VEGF signaling pathway, African trypanosomiasis, Glutamatergic synapse, and Fc epsilon RI signaling pathway (Figure 6D).

## 4 Discussion

Inflammatory bowel disease (IBD) can be classified into ulcerative colitis and Crohn’s disease (CD). Both types present similar symptoms, including diarrhea, intestinal bleeding, abdominal pain, and varying degrees of weight loss. UC primarily affects the colon and rectum (Tontini et al., 2015). The intestinal mucosal barrier serves as the first line of defense against pathogenic organisms. The mucus layer, an essential component of this barrier, contains mucin secreted by goblet cells, which protects the intestinal mucosa from pathogenic bacterial colonization and acid–base erosion (Pelaseyed et al., 2014). Studies have shown that *Pulsatilla* can significantly reduce mesenteric blood flow and the inflammatory response in UC rats (Liu et al., 2021). In this study, both the high-dose and low-dose groups exhibited significantly longer colons than the model group, and both doses improved colonic ulcers in UC rats. These findings suggest that traditional Chinese botanical drugs has a therapeutic effect on DSS-induced UC. Additionally, the intestinal flora plays a crucial role in the onset and progression of UC.

Microbiota sequencing of cecal contents using 16S rDNA in this study revealed reduced alpha diversity in mice with DSS-induced colitis. High-throughput sequencing showed that the dominant phyla in each group were *Firmicutes* and *Bacteroidota*. *Bacteroidetes* and *Firmicutes* constitute the primary intestinal flora in mice, playing key roles in carbohydrate transport and regulation. The severity of intestinal flora imbalance directly affects the progression of inflammation (Coqueiro et al., 2019). Short-chain fatty acids, produced by symbiotic intestinal flora, inhibit the growth of harmful bacteria in the intestinal lumen and play a vital role in protecting intestinal barrier function and regulating immune responses. Studies have shown that SCFAs (short chain fatty acids) are positively correlated with *Firmicutes* and *Actinobacteria* (Fang et al., 2023). Other studies have found that DSS decreases the abundance of *Actinobacteriota* in the mouse gut, while the addition of probiotics or polysaccharides can increase their abundance (Gryaznova et al., 2022; Zhang et al., 2021). Consistent with these findings, the abundance of *Actinobacteriota* in the model group was significantly lower than that in the other groups. We hypothesize that MPD improves the intestinal environment by adjusting the proportion of probiotics, leading to an increase in SCFAs. Therefore, further examination of factors related to the intestinal mucosal barrier and SCFAs content is warranted. Our findings suggest that MPD may serve as a promising adjunct therapy for ulcerative colitis (UC) by regulating the microbiota. The identified mechanisms could inform clinical strategies by: developing MPD herbal formulations targeting gut microbiota, elucidating the microbial regulatory mechanisms of MPD, and clarifying the pathological connection between gut microbiota and UC. Additionally, DSS increased the abundance of *Staphylococcus* in the intestinal tract of the model group. Components of MPD, such as *Pulsatilla chinensis*, *Lonicerae hypoglauca*, *Magnoliae officinalis*, and *Scutellaria baicalensis*, exhibit inhibitory effects on *Staphylococcus*, with different active ingredients suppressing its proliferation through various mechanisms (Dasagrandhi et al., 2018; Huang et al., 2006; Zhao et al., 2018; Zhong et al., 2022). It is speculated that MPD modulates gut microbiota by decreasing *Staphylococcus* levels. *Oscillospirates* were predominant in the high-dose group. Although challenging to culture using traditional microbiological techniques, *Oscillospirates* are consistently detected, and their reduction can lead to inflammation (Konikoff and Gophna, 2016). The abundance of *Oscillospirates*, primarily influenced by exogenous factors, produces butyrate, an essential immunomodulatory molecule in the intestine that is closely related to intestinal homeostasis (Faden, 2022; Su et al., 2014). In conclusion, MPD ameliorates UC in mice by increasing SCFAs and inhibiting the growth of *Staphylococcus*.

Metabolomics, which analyzes an organism’s physiological and pathological status through metabolites, is a vital tool for studying disease mechanisms and drug effects. In this study, traditional Chinese botanical drugs significantly alleviated DSS-induced ulcerative colitis in mice. The results indicate that MPD primarily affects pathways related to linoleic acid metabolism, VEGF signaling, and the glutamatergic synapse. Linoleic acid, an essential unsaturated fatty acid, has been shown to promote the clearance of *Staphylococcus aureus* in macrophages (Gomez-Cortes et al., 2019; Yan et al., 2022). MPD treatment significantly reduced the abundance of *Staphylococcus*, suggesting that MPD may alleviate DSS-induced *Staphylococcus* enrichment in the mouse intestine by regulating linoleic acid metabolism, thereby reducing intestinal inflammation.

## 5 Conclusion

This study provides preliminary evidence that our specific MPD can ameliorate ulcerative colitis in mice, possibly by regulating gut microbiota, increasing short-chain fatty acids, and reducing intestinal inflammation.

## Data Availability

The original contributions presented in the study are included in the article/supplementary material, further inquiries can be directed to the corresponding author.

## Footnotes

^1^ www.majorbio.com

^2^ http://www.hmdb.ca/

^3^ https://metlin.scripps.edu/
